# Human DNA ligase IV is able to use NAD^+^ as an alternative adenylation donor for DNA ends ligation

**DOI:** 10.1093/nar/gky1202

**Published:** 2018-11-28

**Authors:** Shih-Hsun Chen, Xiaochun Yu

**Affiliations:** Department of Cancer Genetics & Epigenetics, Beckman Research Institute, City of Hope, Duarte, CA 91010, USA

## Abstract

All the eukaryotic DNA ligases are known to use adenosine triphosphate (ATP) for DNA ligation. Here, we report that human DNA ligase IV, a key enzyme in DNA double-strand break (DSB) repair, is able to use NAD^+^ as a substrate for double-stranded DNA ligation. In the *in vitro* ligation assays, we show that the recombinant Ligase IV can use both ATP and NAD^+^ for DNA ligation. For NAD^+^-mediated ligation, the BRCA1 C-terminal (BRCT) domain of Ligase IV recognizes NAD^+^ and facilitates the adenylation of Ligase IV, the first step of ligation. Although XRCC4, the functional partner of Ligase IV, is not required for the NAD^+^-mediated adenylation, it regulates the transfer of AMP moiety from Ligase IV to the DNA end. Moreover, cancer-associated mutation in the BRCT domain of Ligase IV disrupts the interaction with NAD^+^, thus abolishes the NAD^+^-mediated adenylation of Ligase IV and DSB ligation. Disrupting the NAD^+^ recognition site in the BRCT domain impairs non-homologous end joining (NHEJ) in cell. Taken together, our study reveals that in addition to ATP, Ligase IV may use NAD^+^ as an alternative adenylation donor for NHEJ repair and maintaining genomic stability.

## INTRODUCTION

DNA ligases, discovered by the Gellert, Lehman, Richardson and Hurwitz laboratories in 1967, are essential enzymes for DNA metabolism, such as DNA replication, repair and recombination (1–3). The molecular mechanism of DNA end ligation mediated by DNA ligases is evolutionarily conserved from prokaryotes to primates. The first step for DNA ligation is to adenylate the ligase with an adenosine monophosphate (AMP) moiety. Subsequently, the AMP moiety is transferred from ligase to the 5′ phosphate group of DNA. The hydroxyl group at 3′ end then attacks the adenylated 5′-phosphate to establish the phosphodiester bond between the two ends for the completion of the ligation.

In eukaryotes, DNA ligases are known to only use adenosine triphosphate (ATP) for the adenylation (4–7). However, ATP is not the only source for adenylation during the evolution of DNA ligation. In prokaryotes, based on the different sources of the AMP moiety in the first step of the ligation, DNA ligases are categorized into two groups, ATP-dependent ligases and NAD^+^-dependent ligases (8–10). For example, T4 DNA ligase is an ATP-dependent ligase (11–15); whereas Ligase A is an NAD^+^-dependent ligase (16,17). DNA ligases in archaea are more diversified as some archaeal ligases are able to use both ATP and NAD^+^ (18–21) or even adenosine diphosphate (ADP) for ligation (22,23). However, to date, all the eukaryotic DNA ligases are known as ATP-dependent ligases (4–7).

Interestingly, most NAD^+^-dependent DNA ligases contain the BRCA1 C-terminal (BRCT) domain. Originally, we and others found that a set of BRCT domain such as the BRCA1 BRCT domain is a phospho-Ser binding domain (24–26). Although the primary sequences are quite different, the similar secondary and tertiary structures of the BRCT domains always harbor a phosphate group binding pocket. Recently, we showed that a few BRCT domains are able to recognize ADP-ribosylation (27,28). Mutating the phosphate group binding pockets abolish the interactions with ADP-ribose (ADPr), indicating that a set of the BRCT domain may recognize the phosphate group in ADPr. One of the BRCT domains that recognize ADPr is the human Ligase IV BRCT domain. Since ADPr is derived from NAD^+^, we wondered if the Ligase IV BRCT domain recognizes NAD^+^, and if Ligase IV may use NAD^+^ as an adenylation donor for DNA ends ligation.

Although Ligase IV is able to repair the nicked DNA, it is a crucial component that functions in DNA non-homologous end joining (NHEJ), a predominant pathway of DNA double-strand break (DSB) repair in mammalian cells (29–31). Mutations of Ligase IV induce Ligase IV syndrome, a rare genetic disorder associated with immunodeficiency, radiation sensitivity and childhood lymphoma (4). Moreover, mutations of Ligase IV are also associated with Dubowitz syndrome (32), Omenn syndrome (33) and radiation-sensitive severe combined immunodeficiency (34,35). In addition to possessing a canonical DNA ligase catalytic domain, Ligase IV is distinct from other eukaryotic DNA ligases in that it possesses a C-terminal BRCT domain with two tandem BRCT motifs. Ligase IV forms a complex with XRCC4 for NHEJ. The interaction with XRCC4 requires the linker region between the two BRCT motifs (36,37). It has been shown that Ligase IV can use ATP for DNA ligation in various organisms (38,39). These Ligase IV orthologs function together with XRCC4 orthologs in NHEJ (38–43). However, since Ligase IV contains a BRCT domain at the C-terminus, we ask if Ligase IV is also able to use NAD^+^ for DNA ligation. Here, we show the evidence that Ligase IV is able to use NAD^+^ for DNA nick or end ligation. The BRCT domain of Ligase IV facilitates the recognition of NAD^+^ toward the adenylation of Ligase IV, the first step of the ligation. The Ligase IV syndrome mutations disrupting the BRCT domain abolishes the adenylation of Ligase IV.

## MATERIALS AND METHODS

### DNA substrates

Oligonucleotides used in this study were synthesized by Integrated DNA Technologies, Inc. and purified by denaturing polyacrylamide gel electrophoresis (PAGE). Oligonucleotides were labeled at the 5′-end with [γ-^32^P]ATP (3000 Ci/mmol) (PerkinElmer, Boston, MA, USA) and T4 polynucleotide kinase (New England Biolabs, Beverly, MA, USA) according to the manufacturer’s instructions. Unincorporated radioisotope was removed by G-25 Sephadex (Amersham Biosciences, Inc., Piscataway, NJ, USA) spin-column chromatography. To generate the double-stranded DNA substrates, labeled oligonucleotides were annealed with the unlabeled complementary oligonucleotide.

**Figure 1.**
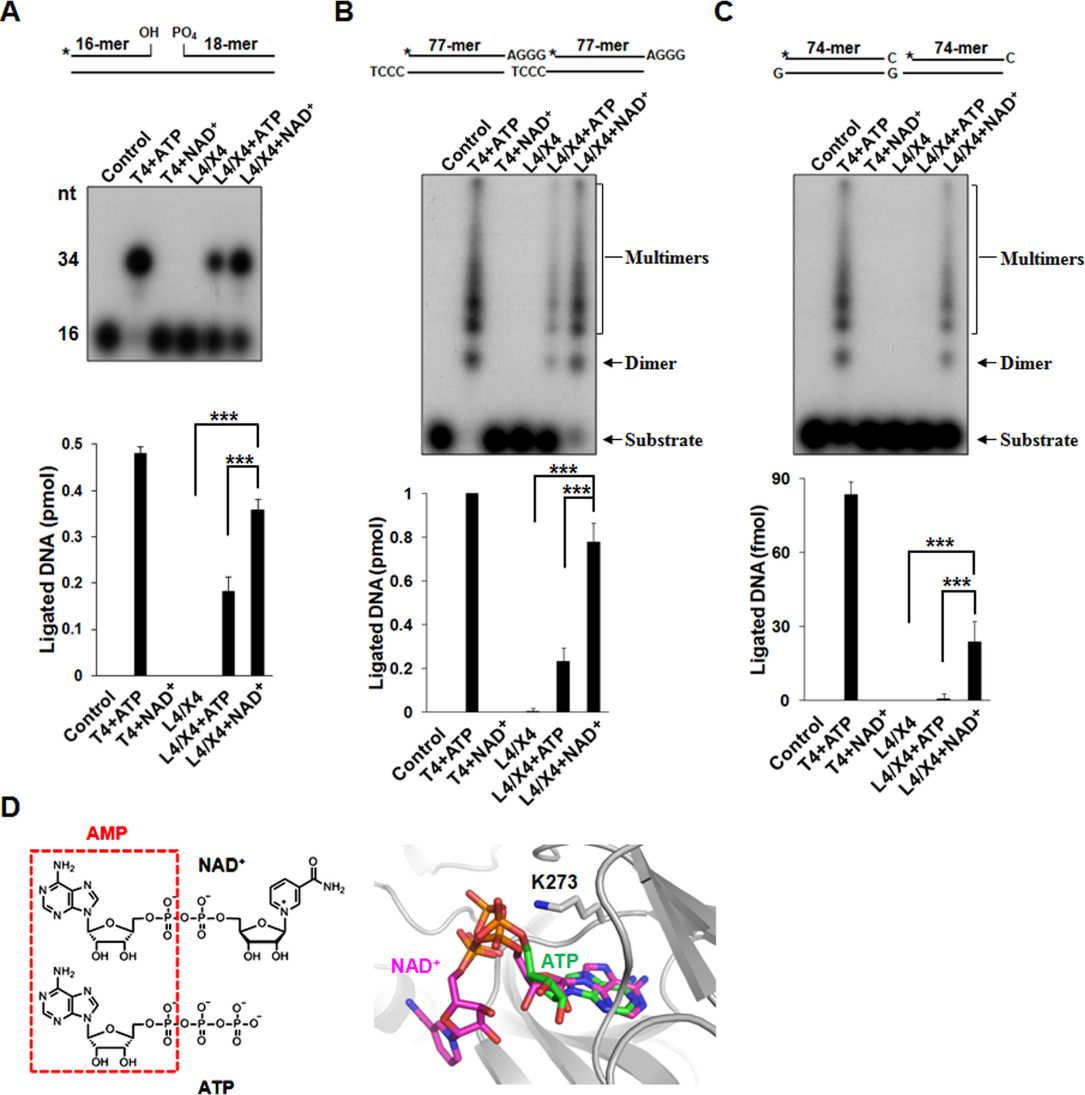
The Ligase IV/XRCC4 complex uses NAD^+^ for DNA ligation, 0.5 pmol (50 nM) Nicked (**A**), 1 pmol (100 nM) 4 nt-overhang (**B**) and 1 pmol (100 nM) 1 nt-overhang (**C**) DNA end substrates were incubated with 25 nM Ligase IV/XRCC4 (L4/X4) complex in 10 μl reaction buffer. One milimolar ATP and NAD^+^ were used as adenylate source. A star indicates the position of the radioisotope label. Ligated products were analyzed and presented as mean ± SD from three independent experiments. Control means no protein, and T4 DNA ligase (T4, 4000 units/ml) with ATP was used as a positive control. L4/X4 means Ligase IV/XRCC4 without any co-factor. *** denotes *P* < 0.001. (**D**) The binding mode of NAD^+^ in the adenylation domain of human DNA Ligase IV is similar with that of ATP (PDB: 3W5O). Both NAD^+^ and ATP contain AMP moiety (left panel); AMP moieties of NAD^+^ and ATP are superimposed (right panel).

For nicked DNA, a 34-nt oligomer (5′-TCAAGTTAGTATGTCA AAGCAGGCTTCAACGGAT-3′) was annealed to two complementary strands, ^32^P-labeled 16-mer (5′-ATCCGTTGAAGCCTGC-3′) and 5′ phosphorylated 18-mer (5′-TTTGACA TACTAACTTGA-3′) oligos, to make a nicked DNA substrate for ligation (Figure 1A). For overhang DNA substrates, we used two 77-nt oligomers (^32^P-labeled 5′-GTTAAGTATCTGCATCTTACTTGACGGATGCAATCGTCACGTGCTAGACTACTGGTCAAGCGGATCGGGCTCGAGGG-3′ and 5′-CGAGCCCGATCCGCT TGACCAGTAGTCTAGCACGTGACGATTGCATCCGTCAAGTAAGATGCAGATACTTAACCCCT-3′) and two 74-nt oligomers (^32^P-labeled 5′-CGAGCC CGATCCGCTTGACCAGTAGTCTAGCACGTGACGATTGCATCCGTCAAGTAAGATGCAGATACTTAACC-3′ and 5′-GTTAAGTATCTGCATCTTACTTGACG GATGCAATCGTCACGTGCTAGACTACTGGTCAAGCGGATCGGGCTCGG-3′) to make 4 nt- and 1 nt-overhang DNA substrates for ligation, respectively (Figure 1B and C). For Figure 3D and E, a substrate with a mismatch at the 3′ end of a nick was formed by annealing 16-mer (5′-ATCCGTTGAAGCCTGT-3′) and ^32^P-labeled 18-mer (5′-TTTGACATACTAACTTGA-3′) oligos to the 34-mer complementary strand (5′-TCAAGTTAGTATGTCAAAGCAGGCTTCAACGG AT-3′).

### Cell culture

Sf9 cells were grown in Grace’s Insect medium supplemented with 10% fetal bovine serum (FBS) and incubated at 27°C. Ligase IV^−/-^ MEFs and U2OS cells were cultured in Dulbecco’s Modified Eagle Medium supplemented with 10% FBS and incubated with 5% CO_2_ at 37°C. Ligase IV^−/-^ MEFs were obtained from Dr Shan Zha at Columbia University.

### Generation of stable cell lines

The complementary DNA of human NAD^+^ Kinase (NADK), wild-type Ligase IV and Ligase IV mutant (S668A) were cloned into pS-Flag-SBP vector. MEFs or U2OS cells were transfected with constructed plasmids using Lipofetamine 2000 (Invitrogen, CA, USA) and were selected by puromycin treatment. Cells stably expressing NADK or Ligase IV constructs were validated by western blotting using anti-NADK antibody (Proteintech group, Inc.) or anti-Ligase IV antibody (GeneTex, Inc.). β-Actin was examined by anti-β-Actin antibody (Sigma-Aldrich) as the protein loading control.

### Protein expression and purification

Ligase IV/XRCC4 and its variants were overexpressed in Sf9 cells using ‘Bac-To-Bac’ Baculovirus expression system (Invitrogen). The GST-tagged Ligase IV and XRCC4 (without tag) were purified by baculovirus-insect cell system and Mono Q column. The recombinant GST-tagged Ligase IV BRCTs and its variants were overexpressed in *Escherichia coli* strain BL-21(DE3). A 10 ml overnight culture of a single transformant was used to inoculate 1 l of fresh LB medium containing 100 μg/ml ampicillin. The cells were grown at 37°C to *A*_600_ = 0.6 and induced with 1 mM isopropyl-β-thiogalactopyranoside for 16 h at 18°C. The cells were harvested by centrifugation at 5000 rpm for 10 min and the pellet was suspended in NETN100 buffer (20 mM Tris–HCl at pH 8.0, 100 mM NaCl, 1 mM ethylenediaminetetraacetic acid (EDTA), 0.5% nonidet P-40). A sonicator instrument was used to disrupt the cells and centrifuged at 12 000 rpm for 20 min to discard the debris. The cell-free extract was incubated with glutathione Sepharose 4B (Amersham) for 2 h at 4°C. The beads were washed with NETN100 buffer and the protein was eluted with 100 mM Tris buffer (pH 8.5) containing 10 mM reduced-glutathione. The eluted proteins were dialyzed against elution buffer to remove reduced-glutathione. The protein concentrations were determined by the Bradford method (BioRad, USA). The purified proteins were confirmed by sodium dodecyl sulphate (SDS)-PAGE and stored in Phosphate buffered saline (PBS) at −80°C.

### Adenylation

Reaction mixtures (10 μl) containing 50 mM Tris–HCl (pH 8.0), 5 mM dithiothreitol (DTT), 10 mM MgCl_2_, 1.5 μM ATP or NAD^+^ (1% [α-^32^P] ATP or ^32^P-NAD^+^) and 25 nM enzyme as specified were incubated for 30 min at 25°C. The reactions were quenched at 95°C in SDS buffer and the products were analyzed by 7.5% SDS-PAGE. The covalent ligase–[^32^P]AMP complex was visualized by autoradiography.

### DNA ligation assay

To remove the endogenous adenylation, Pyrophosphate pretreatment was performed in 50 mM Tris–HCl (pH 8.0), 5 mM DTT and 10 mM MgCl_2_ for 15 min at 25°C with 5 mM Na_2_P_2_O_7_ (Sigma-Aldrich) and removed residual PPi by desalting column (Millipore). Nick ligation was done in a 10 μl reaction. Ligation buffer contains 50 mM Tris–HCl (pH 8.0), 10 mM MgCl_2_, 5 mM DTT and 1 mM cofactor. Ligations were carried out with 25 nM Ligase IV/XRCC4 complex and 0.05 μM (0.5 pmol) nicked DNA end substrate (a 34-nt bottom strand annealed to a complementary ^32^P-labeled 16-mer and a 5′-phosphorylated 18-mer forming the nicked top strand). Ligations were incubated for 1 h at 25°C, denatured at 95°C in formamide and fractionated on 15% TBE-Urea gels. Ligation activity was analyzed by autoradiography.

The overhang DNA ligation assay was performed in a 10 μl reaction. 0.1 μM (1 pmol) ^32^P-labeled DNA end substrates were first incubated with 25 nM Ku70/80 (Protein Specialists, NJ, USA) in the ligation buffer (50 mM Tris–HCl (pH 8.0), 10 mM DTT, 10% polyethylene glycol (PEG10000), and 50 μg/ml bovine serum albumin) at room temperature for 15 min. Ligation was initiated by adding 10 mM MgCl_2_ and 1 mM cofactor (for NAD^+^, 1, 0.1 or 0.2 mM) with 25 nM Ligase IV/XRCC4 complex. Reactions were then incubated at 25°C for 2 h. After incubation, reactions were stopped at 95°C in formamide. Ligation results were analyzed by 10% TBE-Urea gels and autoradiography.

### Calculation of kinetic parameters

Kinetic analysis of multiple turnover reaction was performed in the identical ligation buffer of overhang DNA ligation assay with 25 nM Ligase IV/XRCC4 and 500 nM DNA end substrates. The concentrations of NAD^+^ were used from 0.156 μM to 5 μM. Reactions were carried out for 0.5 h at 37 °C. Kinetic analysis of single turnover reaction was performed in the identical ligation buffer of overhang DNA ligation assay with 500 nM Ligase IV/XRCC4 and 100 nM DNA end substrates. The concentrations of ATP were used from 0.3125 to 10 μM. Reactions were carried out for 1 min at 37 °C. All reactions were stopped at 95°C in formamide. Samples were analyzed by 10% TBE-Urea gels and autoradiography. Ligation efficiency was estimated by Image J. The multiple-turnover kinetic parameters were calculated according to Michaelis–Menten equation. The single-turnover parameters were calculated according to previous study (44).

### GST pull-down and thin-layer chromatograph (TLC) analysis

Recombinant GST-tagged Ligase IV BRCTs or its variants (1 μM) was incubated with 500 nM NAD^+^ or ATP (10 nM ^32^P-NAD^+^ or ^32^P-ATP and 490 nM unlabeled NAD^+^ or ATP) for 30 min at 4°C. Mixture (100 μl) was bound to glutathione Sepharose 4B (Amersham) for 2 h at 4°C and then washed at least three times with PBS. The samples were heated at 95°C for 5 min and examined by thin-layer chromatograph (TLC). Samples was spotted on cellulose TLC plates (Merck) and developed with 0.9 M Acetic acid and 0.3 M lithium chloride. Plates were dried and analyzed by autoradiography.

### App-DNA formation assay

After adenylation with 1.5 μM NAD^+^ or ATP (1% ^32^P-NAD^+^ or ^32^P-ATP) for 30 min at 25°C, adenylated Ligase IV complexes were incubated with and without 5 nM ^32^P-labeled 3′ mismatch DNA and 1 mM NAD^+^ or ATP for 1 h at 25°C. The reactions were stopped at 95°C in formamide. App-DNA formation was analyzed by 20% TBE-native gel and autoradiography.

### Modeling and docking analysis

Molecular docking of NAD^+^, NADP^+^ or ATP with human DNA Ligase IV (PDB: 3W5O) or Ligase IV BRCT domain (PDB: 2E2W) was evaluated using GEMDOCK (45) software. Intermolecular interactions between docked NAD^+^ and protein residues as well as comparisons of active sites of Ligase IV (PDB: 3W5O) and other DNA ligases (PDB: 2OWO, 1TAE, 1ZAU, 3JSN, 1 × 9N and 3L2P) were analyzed using Swiss-PdbViewer 3.7 software. The images were revealed by PyMoL and Swiss-PdbViewer 3.7 softwares.

### NHEJ assay

The NHEJ reporter construct was kindly provided by Dr Vera Gorbunova (University of Rochester). The schematic of the construct and procedure of the report assays are illustrated in Figure 5A. For the NHEJ reporter assay, the NHEJ reporter construct was digested overnight with HindIII at 37°C and gel purified. The pre-digested construct was transfected into cells. After 72 h transfection, cells were harvested for FACS analysis. The percentage of green fluorescent protein (GFP)-positive cells (successful NHEJ repair) was calculated.

### The measurement of NADP^+^/NAD^+^ ratio

The cytoplasmic and nuclear extracts from U2OS control cells (vector only) and U2OS-NADK cells were obtained using the NE-PER Nuclear and Cytoplasmic Extraction Reagents kit (Thermo Fisher Scientific) according to the manufacturer's protocol. The cytoplasmic and nuclear concentrations of NAD^+^ and NADP^+^ were estimated using EnzyChrom™ assay Kits. The concoction was read optical density at 570 nm by ELISA reader. Sample NAD^+^ and NADP^+^ concentrations were determined from the standard curve.

### Comet assay

Following IR (5 Gy) treatment, cells were collected and rinsed twice with ice cold PBS. 1 × 10^5^ cells/ml were combined with 1% LMAgarose at 37°C at a ratio of 1: 10 (v/v) and immediately pipetted onto slides. For cell lysis, the slides were immersed in the neutral lysis solution (2% sarkosyl, 0.5 M EDTA, 0.5 mg/ml proteinase K, pH 8.0) overnight at 37°C in dark, followed by washing in the rinse buffer (90 mM Tris buffer, 90 mM boric acid, 2 mM Na_2_EDTA in pH 8.5) for 30 min with two repeats. Then, the slides were subjected to electrophoresis at 20 V for 25 min (0.6 V/cm), and stained in 2.5 μg/ml propidium iodide for 20 min. Images were taken with a fluorescence microscope.

### Immunofluorescence

Cells were treated with 2 Gy IR for γ-H2AX foci formation assay and recovery in fresh media at 37°C. Treated cells were fixed in 3% paraformaldehyde for 15 min at room temperature and permeabilized in 20 mM 4-(2-hydroxyethyl)-1-piperazineethanesulfonic acid (HEPES) (pH 7.4), 50 mM NaCl, 3 mM MgCl_2_, 300 mM sucrose and 0.5% Triton X-100 (Sigma-Aldrich) for 15 min at 4°C. After blocking with 5% goat serum (Sigma-Aldrich) in PBS, anti-γ-H2AX antibody (1:500, Millipore) incubations were performed for overnight at 4°C and followed by washing in PBS. Incubations with anti-mouse FITC secondary antibodies (Sigma-Aldrich) were performed at room temperature at 1:500 dilutions in 5% goat serum for 1 h in the dark. Nuclei were counterstained with 4′,6-diamidino-2-phenylindole (Sigma-Aldrich) for 10 min at room temperature in the dark. Coverslips were mounted in Vectashield (Vector Laboratories, Peterborough, United Kingdom). Results were analyzed using a fluorescence microscope.

### Metaphase spreads

Following IR (1 Gy) treatment, cells were treated with colcemid (1 μg/ml) at 37°C for 2 h. Cell lysate were harvested and incubated in 7 ml 0.56% KCl solution at room temperature for 10 min. KCl was discarded from the supernatant after centrifugation at 1000 rpm for 10 min. The cells were fixed in a fresh, cool fixative (3 methanol: 1 glacial acetic acid) gradually increased to 7 ml before centrifugation again at 1000 rpm for 10 min; then the supernatant was discarded. The cells were fixed at least three times; then the pellet was mixed with 1 ml fixative. The mixture was dropped onto a clean and cold slide by a micropipette followed by an air-dry technique. Samples were stained with 4′,6-diamidino-2-phenylindole (DAPI) and analyzed by microscope.

### Cell viability analysis

Cells were seeded into six-well plates (∼1000 cells/well) and treated by IR (1, 2, 4, 8 Gy) or MMS (75, 150, 300 μM for 2 h) treatment. After a 14-d culture, the viable cells were fixed by methanol and stained with crystal violet. The number of colonies (>50 cells for each colony) was calculated.

## RESULTS

### Ligase IV can use NAD^+^ for DNA ligation

To examine if Ligase IV is able to use NAD^+^ for DNA end ligation, we generated the recombinant Ligase IV/XRCC4 complex, removed pre-existed adenylation in the complex and performed DNA nick ligation and DSB ligation *in vitro* with either ATP or NAD^+^. With T4 DNA ligase as the positive control, we found that both of ATP and NAD^+^ could be utilized by the Ligase IV/XRCC4 complex to repair the nicked DNA. Notably, more ligated DNA products were observed when NAD^+^ was used in this assay (Figure 1A). Similar results were obtained from a time-course nick ligation assay (Supplementary Figure S1A). For double-stranded DNA ligation, 4 nt- and 1 nt-overhang DNA were labeled with ^32^P and examined in the ligation assays. Again, the ligated products were increased in the NAD^+^-mediated ligation (Figure 1B and C), indicating that the Ligase IV/XRCC4 complex may favor NAD^+^ for the end joining at least *in vitro*. Interestingly, we noticed that more than 80% of the 4 nt-overhang DNA substrates were ligated in the presence of NAD^+^, suggesting that Ligase IV is able to use NAD^+^ to catalyze more than one round of DNA ligation. In contrast, <25% of the 4 nt-overhang DNA substrates were ligated in the presence of ATP, suggesting that ATP-dependent Ligase IV may only catalyze single round of ligation. It is in agreement with earlier studies showing that Ligase IV can only catalyze one round ligation in the presence of ATP (46,47).

To examine if NAD^+^-mediated ligation is Ku-dependent DNA end joining, we performed 4 nt-overhang DNA ligation assays with or without the Ku70/80 complex and found that the Ku70/80 complex promoted DNA ligation (Supplementary Figure S1B). Moreover, we measured the nuclear concentration of NAD^+^ as ∼0.14 mM, which is similar with previous studies (48,49). It has also been reported that the best efficiency of ATP-mediated ligation is with ∼1 mM ATP (50,51). Thus, we performed 4 nt-overhang DNA ligation assays with 1 mM ATP and 0.1 or 0.2 mM NAD^+^, which is close to the physiologically relevant levels of ATP or NAD^+^ during ligation (Supplementary Figure S1C). Again, we found that NAD^+^ promotes Ligase IV-dependent ligation. In addition, we incubated 20 nM adenylated Ligase IV with 100 nM DNA end substrates for one round ligation, and then added additional ATP or NAD^+^ to promote the second round ligation in the *in vitro* ligation. Our results show that NAD^+^ could be used for DNA ligation in the second round of DNA end joining, suggesting DNA ligase IV is a multiple turnover enzyme with NAD^+^ (Supplementary Figure S1D).

As the detailed structure of the enzymatic domain of Ligase IV has been solved, we performed docking analyses with NAD^+^ or NADP^+^. Like ATP, NAD^+^ could be superimposed well in the catalytic cage (PDB: 3W5O (52)). In particular, NAD^+^ is able to be exposed to Lys273, the key residue to form ligase-adenylate complex (Figure 1D). when NADP^+^ is superimposed into the catalytic site of Ligase IV, the extra phosphate at the 2′ position of ribose sugar favors to locate in a positively charge environment consist of Lys293, Arg443 and Arg449, which causes long distance (>5Å) between diphosphate and Lys273, thus impairs any interaction (Supplementary Figure S2). Thus, collectively these results suggest that human DNA Ligase IV is able to use NAD^+^ as an alternative cofactor to ligate DNA ends.

### Kinetics of NAD^+^-mediated ligation

Next, we measured the kinetic parameters of NAD^+^-mediated ligation by the Ligase IV/XRCC4 complex under steady-state and multiple turnover condition. The kinetics parameters including *K*_m_, *k*_cat_ and *k*_cat_/*K*_m_ were obtained by running concentration series of NAD^+^, which was fit into Michaelis–Menten equation. a *K*_m_ of 0.51 ± 0.12 μM and a *k*_cat_/*K*_m_ of 1.7 ± 0.7 × 10^4^ M^−1^S^−1^ (a *k*_cat_ of 0.53 ± 0.33 min^−1^) were measured (Supplementary Figure S3A). Moreover, ATP-mediated ligation was measured under single turnover condition (53). On average, the *K*_d,app_ and *k*_lig_/*K*_d,app_ of ATP-mediated ligation were 1.41 ± 0.05 μM and 9.2 ± 0.5 × 10^3^ M^−1^S^−1^ (representing a *k*_lig_ of 0.013 ± 0.001 s^−1^), respectively (Supplementary Figure S3B). Of note, the constants of single-turnover kinetics cannot be directly compared to the Michaelis–Menten parameters. However, these results indicate that Ligase IV may use NAD^+^ for multiple-round ligation at least *in vitro*.

### The BRCT domain of Ligase IV is required for the NAD^+^-mediated ligation

**Figure 2.**
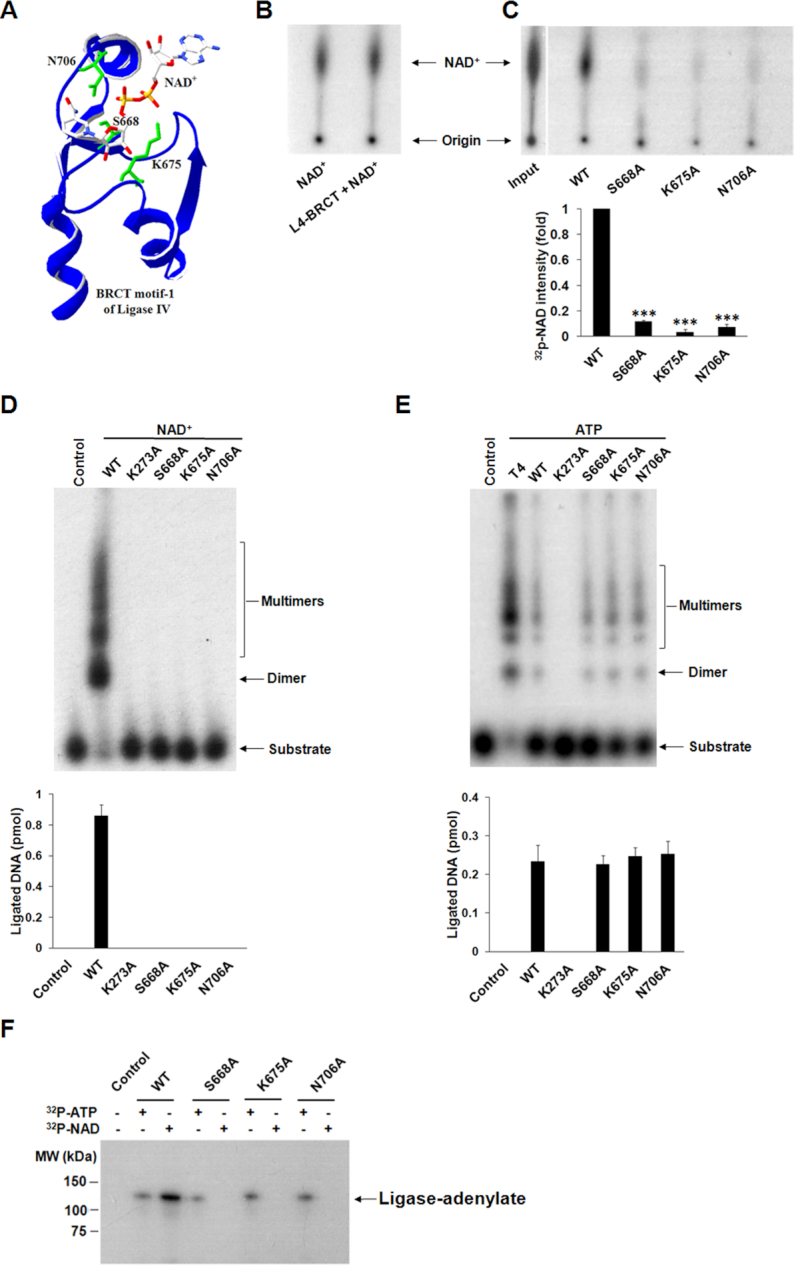
The BRCT domain is required for the NAD^+^-mediated ligation. (**A**) The binding mode of NAD^+^ in Ligase IV BRCT1 is predicted by GEMDOCK and the structure of the BRCT domain of Ligase IV (PDB: 2E2W). Three important residues in the BRCT domain of Ligase IV for the interactions with NAD^+^ are shown in green. (**B** and **C**) The wild-type BRCT domain interacts with NAD^+^. One micromolar wild-type (WT) BRCT domain or three variants were incubated with 500 nM NAD^+^ (10 nM ^32^P-NAD^+^ and 490 nM unlabeled NAD^+^) in the pull-down assays with 100 μl volume. The samples were analyzed by TLC. Relative ^32^P-NAD^+^ intensity compared to WT was presented as mean ± SD from three independent experiments. *** denotes *P* < 0.001. (**D** and **E**) The impact of the BRCT domain for the NAD^+^- and ATP-mediated ligation. The enzymatic activities of the three variants of Ligase IV were examined in the ligation assays with 4 nt-overhang DNA. Control means no protein. The K273A is a catalytic dead form of Ligase IV and was used as a negative control. Bar graphs show the mean of ligated products ± SD from three independent experiments. (**F**) Mutations in the BRCT domain abolish NAD^+^-mediated adenylation, but not ATP-mediated adenylation. Ligase IV wild-type (WT) and three mutants (25 nM) were incubated with 1.5 μM ATP or NAD^+^ (1% α-^32^P-ATP or ^32^P-NAD^+^) in 10 μl reaction buffer. The ligase-adenylate complexes were analyzed by 7.5% SDS-PAGE and autoradiography.

Ligase IV has two tandem BRCT motifs that form a BRCT domain at the C-terminus. Like other BRCT domains, the Ligase IV BRCT contains a putative phosphate-binding pocket. Our previous study shows that the Ligase IV BRCT is able to recognize ADP-ribosylation, which mediates the early recruitment of Ligase IV to DNA lesions (28). As ADPr, the basic unit for ADP-ribosylation, is derived from NAD^+^, we ask if the BRCT domain of Ligase IV is able to recognize NAD^+^ and participates in NAD^+^-mediated ligation. Based on the solved structure (PDB: 2E2W), we performed docking analyses to search for possible interactions between the BRCT domain and NAD^+^ with focusing on the putative phosphate-binding pocket in the BRCT domain. The results show that the α phosphate moiety of NAD^+^ could form electrostatic force/ hydrogen bonds with Lys675; the side chain of Ser668 may have the contact with β phosphate moiety of NAD^+^; and the amino group of nicotinamide would interact with Asn706 by hydrogen bonds (Figure 2A). We also used ATP as a docking control and found it is unable to bind to the pocket surrounded by Ser668 and Lys675 (Supplementary Figure S4A). To validate the analyses, we incubated GST-tagged Ligase IV BRCT domain (residues 654–911) (Supplementary Figure S5) with ^32^P-NAD^+^ and carried out GST-pull down assay and TLC analysis to detect the Ligase IV BRCT domain bound NAD^+^. With ^32^P-NAD^+^ as the loading marker, we found that only the wild-type BRCT domain, but not the K675A, S668A or N706A variants, interacted with NAD^+^ (Figure 2B and C). The results indicate that K675, S668 and N706 play an important role for the recognition of NAD^+^. Moreover, K675A and S668A mutations are in the first BRCT domain of Ligase IV, which are far away from the XRCC4-interaction region. Thus, these mutations do not affect the folding of the BRCT domain, and still form protein complex with XRCC4 (Supplementary Figure S6). However, with either the K675A or S668A mutation, the Ligase IV/XRCC4 failed to ligate DNA ends (Figure 2D). In line with the docking analysis, Ligase IV BRCT domain did not bind ATP when it was incubated with [α-^32^P]-ATP (Supplementary Figure S4B). Additionally, three mutations in the BRCT domain of Ligase IV did not affect ATP-mediated adenylation and ligation (Figure 2E and F). These results suggest that the recognition of NAD^+^ via the phosphate-binding pocket in the BRCT domain plays an important role for Ligase IV-dependent ligation.

### The BRCT domain is required for the NAD^+^-mediated adenylation of Ligase IV

**Figure 3.**
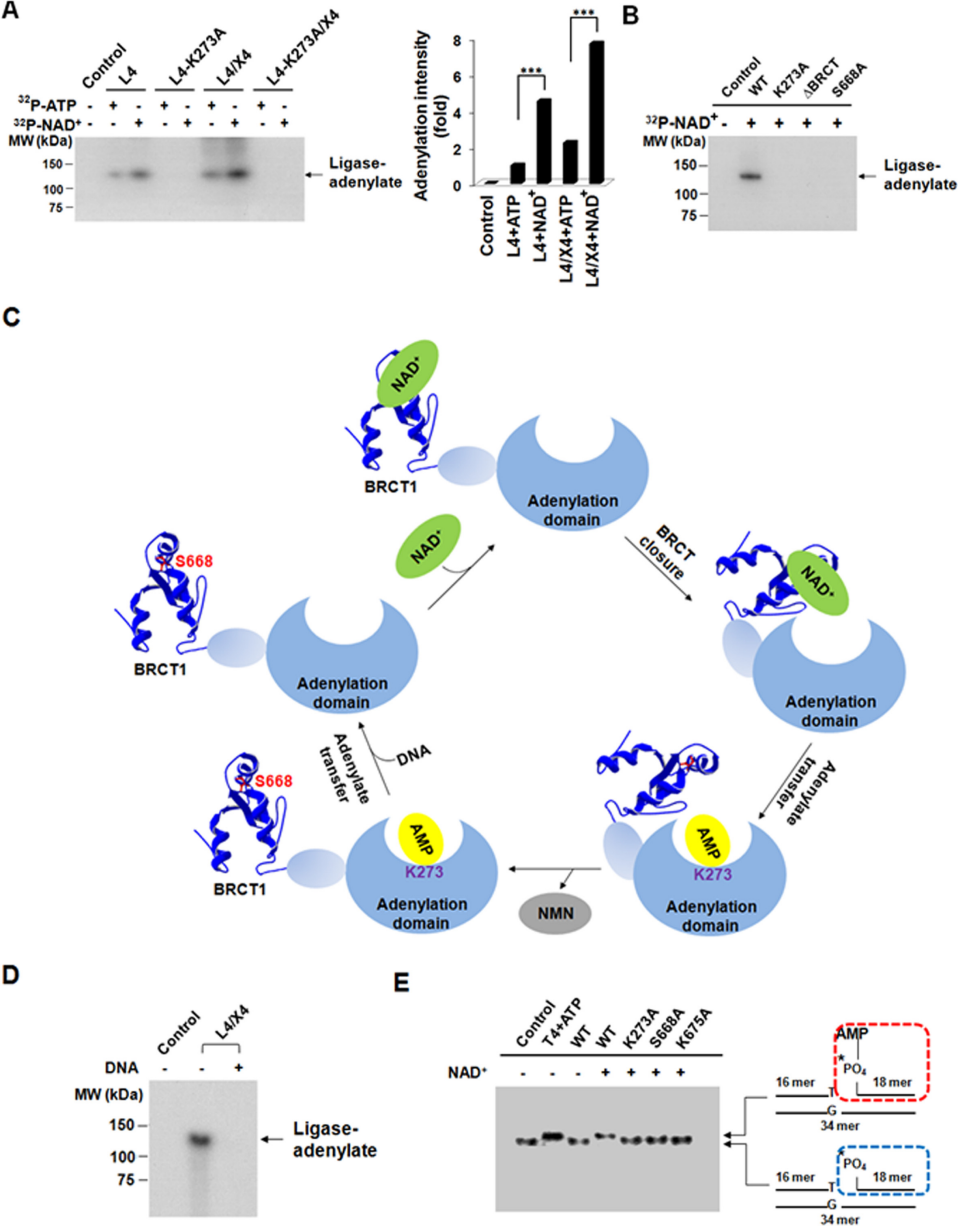
The BRCT domain is important for the NAD^+^-mediated adenylation of Ligase IV. (**A**) The adenylation of Ligase IV. Twenty-five nanomolar Ligase IV (L4) or Ligase IV complex (L4/X4) was incubated with 1.5 μM ATP or NAD^+^ (1% α-^32^P-ATP or ^32^P-NAD^+^) in 10 μl reaction buffer. Samples were analyzed by 7.5% SDS-PAGE and autoradiography. Control means no protein and the K273A mutant was used as a negative control. Bar graph shows the mean of relative ^32^P-AMP-ligase intensity compared to ATP-mediated adenylation (*n* = 3 independent experiments). *** denotes *P* < 0.001. (**B**) Loss of the BRCT domain abolishes the NAD^+^-mediated adenylation. The BRCT domain deletion mutant and the Ser668Ala mutant were examined in the NAD^+^-mediated adenylation assays. (**C**) A model of the NAD^+^-mediated adenylation. (**D**) AMP moiety from NAD^+^ is transferred from Ligase IV to DNA substrates. Twenty-five nanomolar Ligase IV complex was adenylated by ^32^P-NAD^+^, and then incubated with or without 100 nM DNA end substrates in 10 μl reaction buffer. Control means no protein. (**E**) Adenylated DNA (App-DNA) is catalyzed by the Ligase IV complex. The wild-type Ligase IV or its mutants (25 nM) were incubated with 1 mM NAD^+^ for adenylation, then incubated with 5 nM ^32^P-labled 3′ mismatch DNA substrates in 10 μl. A star indicates the position of the radioisotope label. App-DNA complexes were analyzed by 20% TBE-native gel and autoradiography. Control means no protein. T4 DNA ligase (T4, 4000 units/ml) with ATP and the K273A mutant complex (K273A) were used as positive and negative control, respectively.

The first step of DNA ligation is ligase-adenylate formation. To investigate whether NAD^+^ could contribute AMP moiety to form Ligase IV-adenylate complex, we performed the adenylation assay using α-^32^P-ATP or ^32^P-NAD^+^. Consistent with earlier analyses, Ligase IV favored to use NAD^+^ over ATP for adenylation (Figure 3A). Moreover, our results show that XRCC4 is not required for adenylation. To further study the NAD^+^-mediated adenylation, we examined the site of adenylation in the N-terminal catalytic domain of Ligase IV. It has been shown that the adenylation site on Ligase IV is on Lys273 (54). Mutating this residue into alanine (K273A) abolished the adenylation (Figure 3A). Moreover, BRCT-deleted and S668A mutations also abolished NAD^+^-mediated adenylation (Figure 3B), suggesting that the recognition of NAD^+^ via the BRCT domain plays a key role for the first step of DNA ligation. But mutations in the BRCT domain did not affect ATP-mediated adenylation as the BRCT domain is an NAD^+^ recognition motif (Figure 2F). Based on the study, we propose a model for NAD^+^-mediated adenylation (Figure 3C): (i) NAD^+^ is recognized by the phosphate-binding pocket in the Ligase IV BRCT domain, which is within the proximity of the catalytic domain of Ligase IV; (ii) Then Lys273 in catalytic domain attacks NAD^+^ to generate a ligase-adenylate complex and release nicotinamide mononucleotide (NMN); (iii) Adenylated Ligase IV associates with XRCC4 to recognize DNA end and transfers AMP from Lys273 to the 5′-phosphate of DNA. Ligase IV could be re-adenylated by NAD^+^ for the next cycle of ligation.

Following adenylation at Lys273, AMP residue is subsequently transferred to the 5′-phosphate end of DNA to form a DNA-adenylate complex (App-DNA). To test if AMP residue from NAD^+^ could be transferred in the second step of ligation, we included DNA substrates in the adenylation assay, and found that AMP was able to be removed from Ligase IV (Figure 3D) and transferred to DNA (Figure 3E). Moreover, DNA-adenylate complex could not be observed in the presence of the S668A and K675A mutations as it disrupts the interaction with NAD^+^ (Figure 3E). However, these two mutants did not impair the formation of ATP-mediated App-DNA (Supplementary Figure S7). Taken together, it demonstrates that the AMP residue from NAD^+^ can be transferred from the catalytic lysine to the 5′-phosphate of DNA for sequent ligation.

### The mutations of Ligase IV syndrome abolish the interaction with NAD^+^

**Figure 4.**
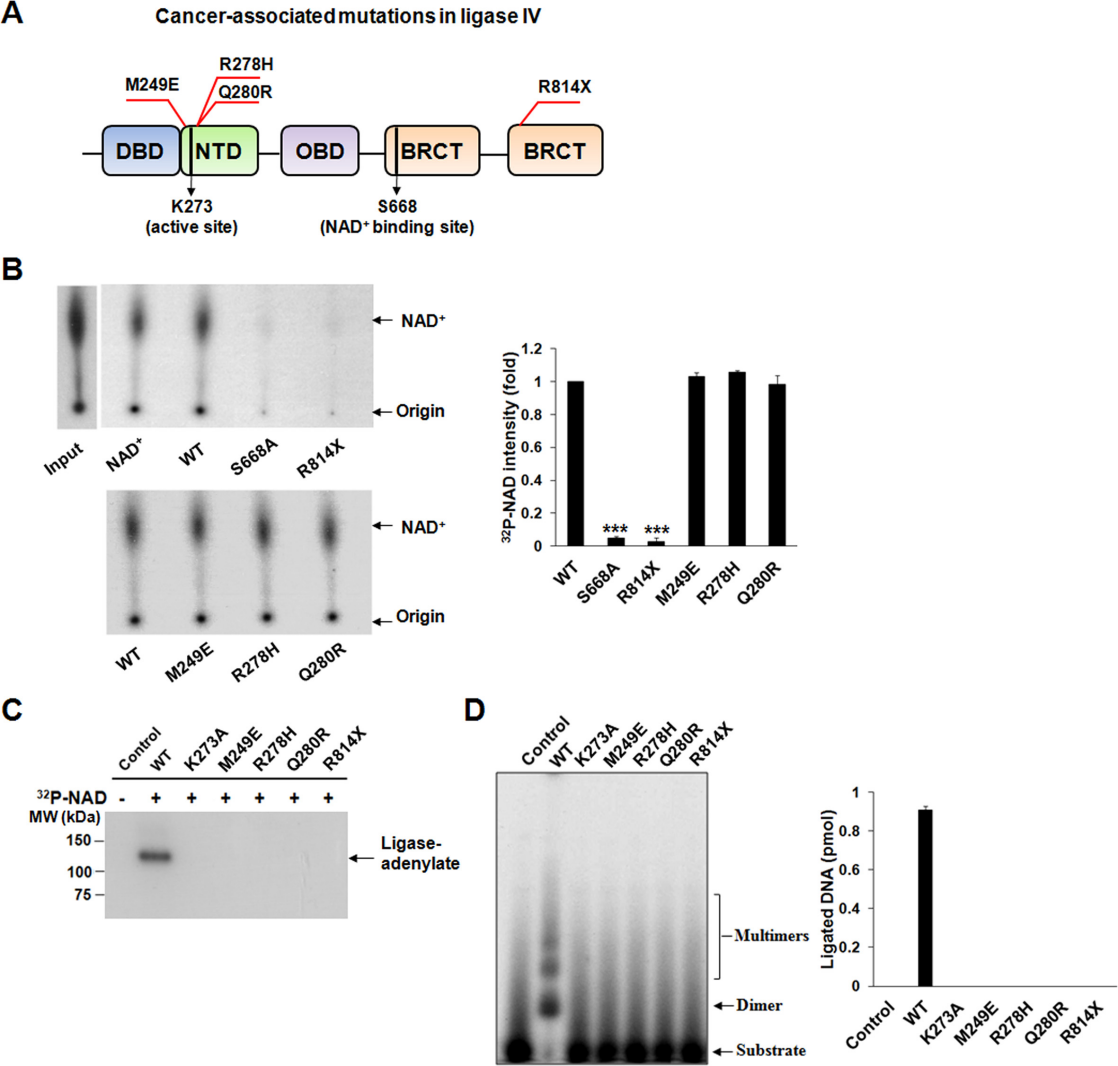
Characterization of cancer-associated mutations of Ligase IV. (**A**) Localization of Ligase IV syndrome mutations. Most mutations (e.g. M249E, R278H and Q280R) localize close to the active site of Ligase IV, whereas one truncating mutation (R814X) is in the BRCT domain. K273 and S668 are in the active site and NAD^+^-binding site of Ligase IV, respectively. (**B**) The R814X abolishes the interaction with NAD^+^, but M249E, R278H and Q280R do not affect the binding of NAD^+^ in BRCT domain. The BRCT domain and its variants (1 μM) were incubated with 0.5 μM NAD^+^ (10 nM ^32^P-NAD^+^). The interaction was examined by the pulled down and TLC assays. ^32^P-NAD^+^ (NAD^+^) was used as a marker. S668A mutant was used as a negative control. Relative ^32^P-NAD^+^ intensity compared to WT were presented as mean ± SD from three independent experiments. *** denotes *P* < 0.001. (**C**) Adenylation is abolished in the cancer-associated Ligase IV mutants. (**D**) NAD^+^-mediated ligation is abolished in Ligase IV mutants. Ligated products were shown as mean ± SD from three independent experiments. In (C) and (D), control means no protein. Wild-type (WT) Ligase IV and the K273A mutant were used as a positive control and negative control, respectively.

Ligase IV plays a key role to repair DSBs and maintain genomic stability, especially during V(D)J recombination. Mutations of Ligase IV induces Ligase IV syndrome with clinical manifestations including severe immunodeficiency, growth retardation and T-cell leukemia. Most Ligase IV syndrome-associated mutations are existed in the catalytic domain. And these mutations, such as M249E, R278H and Q280R, are hypomorphic mutations that are close to Lys273 (Figure 4A), the adenylation site of Ligase IV, thus severely impair the adenylation of Ligase IV and reduce the ligase activity (55). Besides these mutations, it is interesting to note that the BRCT domain also harbors a pathogenic mutation R814X (Figure 4A). The R841X mutation causes a truncation product that deletes the second BRCT motif. It also disrupts the interaction with XRCC4 and abolishes the ligase activity. However, XRCC4 is mainly involved in the last step of ligation, but is not required for the NAD^+^-mediated adenylation of Ligase IV. Since the R814 mutation deletes the second BRCT motif and may abolish the tertiary structure of the BRCT domain, we ask if this mutation in the BRCT domain affects the adenylation, the beginning step of the ligation. We generated the recombinant protein of R814X and examined the interaction between the mutant and NAD^+^. The pull-down and TLC assays show that unlike other catalytic domain mutations, the R841X abolished the interaction with NAD^+^ (Figure 4B), thus abolished the NAD^+^-mediated adenylation (Figure 4C) and ligation (Figure 4D) of Ligase IV. Taken together, the results show that mutations in the BRCT domain induce Ligase IV syndrome through different molecular mechanism.

### The role of NAD^+^-mediated ligation in NHEJ

**Figure 5.**
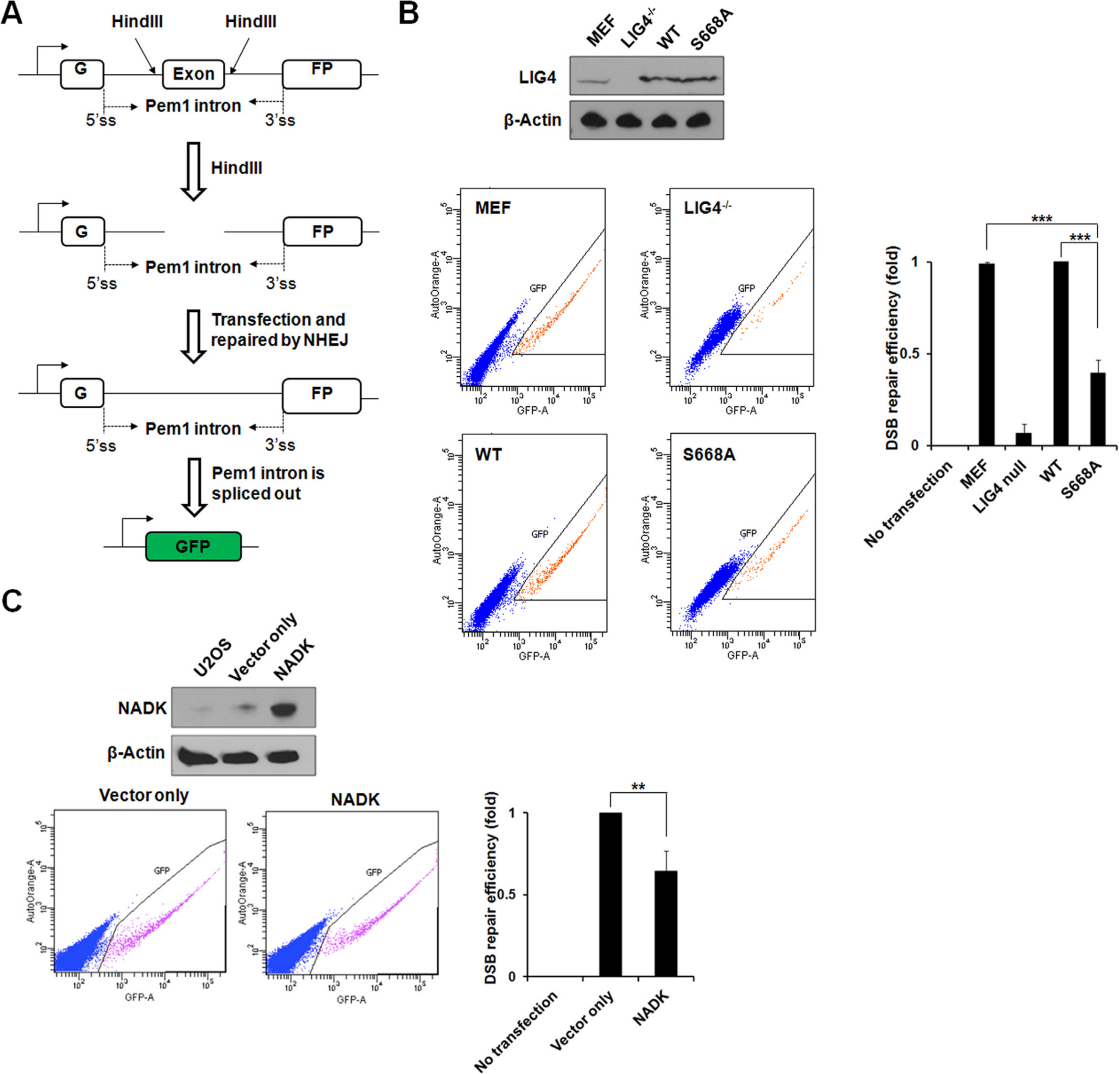
The NAD^+^-mediated Ligase IV plays an important role in NHEJ repair. (**A**) Schematics of NHEJ reporter system. The GFP coding sequence was interrupted by inserting an adenoviral exon flanked by artificial introns. The GFP is inactive due to the expression of the adeno exon in GFP gene; however, once digested with HindIII, the exon will be cut out and a DSB will be created. Successful repair of this DSB using NHEJ will restore the expression of GFP (adapted from Seluanov *etal.*(56)). (**B**) Loss of NAD^+^-mediated ligation suppresses the NHEJ repair. Ligase IV^−/−^ MEFs were reconstituted with wild-type Ligase IV or the S668A mutant. Wild-type MEFs and Ligase IV^−/-^ MEFs (LIG4 null) were used as controls. The number of FACS-sorted GFP+ cells indicates NHEJ repair efficiency and is normalized to NHEJ reporter–transfected cells. Blue dots and orange dots denote GFP- cells and GFP+ cells, respectively. (**C**) Overexpression of NADK suppresses NHEJ repair. U2OS cells expressed NADK to convert NAD^+^ to NADP^+^. NHEJ was examined. Blue dots and pink dots denote GFP- cells and GFP+ cells, respectively. In (B) and C), data were obtained from three independent experiments and presented as mean ± SD. ** denotes *P* < 0.01, *** denotes *P* < 0.001.

The ligase IV-dependent DNA relegation is the last key step of NHEJ, a major mechanism for DSB repair. To characterize the role of NAD^+^-mediated DNA relegation in NHEJ, we assessed NHEJ using a previously described reporter assay, in which GFP is inactive as a result of the insertion of an adenoviral exon flanked by artificial introns in the GFP coding sequence (56). DSBs can be generated in this insertion using a restriction enzyme (Hind III) and the pre-digested construct can be transfected into cells. Successful repair of DSBs with NHEJ restores the expression of GFP. Thus, the percentage of GFP-positive cells is an indicator of successful repair by NHEJ (Figure 5A). We transfected the pre-digested reporter vector into Ligase IV-deficient MEFs stably expressing wild-type Ligase IV or the S668A mutant that abolishes the interaction with NAD^+^. Compared with the wild-type, the S668A mutant significantly reduced the efficiency of NHEJ-mediated DSB repair (Figure 5B), suggesting that the NAD^+^ recognition by the BRCT domain plays an important role in NHEJ. To further validate the results, we used U2OS cells stably expressing NADK, an enzyme phosphorylating NAD^+^ to NADP^+^. Different from NAD^+^, NADP^+^ cannot be used by Ligase IV for end-joining (Supplementary Figure S8). The expression of NADK in nucleus did not affect the cytoplasmic level of NAD^+^, but converted nuclear NAD^+^ to NADP^+^ (Supplementary Figure S9). With the downregulation of NAD^+^ and upregulation of NADP^+^ in nucleus, NHEJ was suppressed (Figure 5C). Collectively, our study reveals that NAD^+^ is also to adenylate Ligase IV and participates in NHEJ.

### NAD^+^-mediated ligation plays an important role in DSB repair

**Figure 6.**
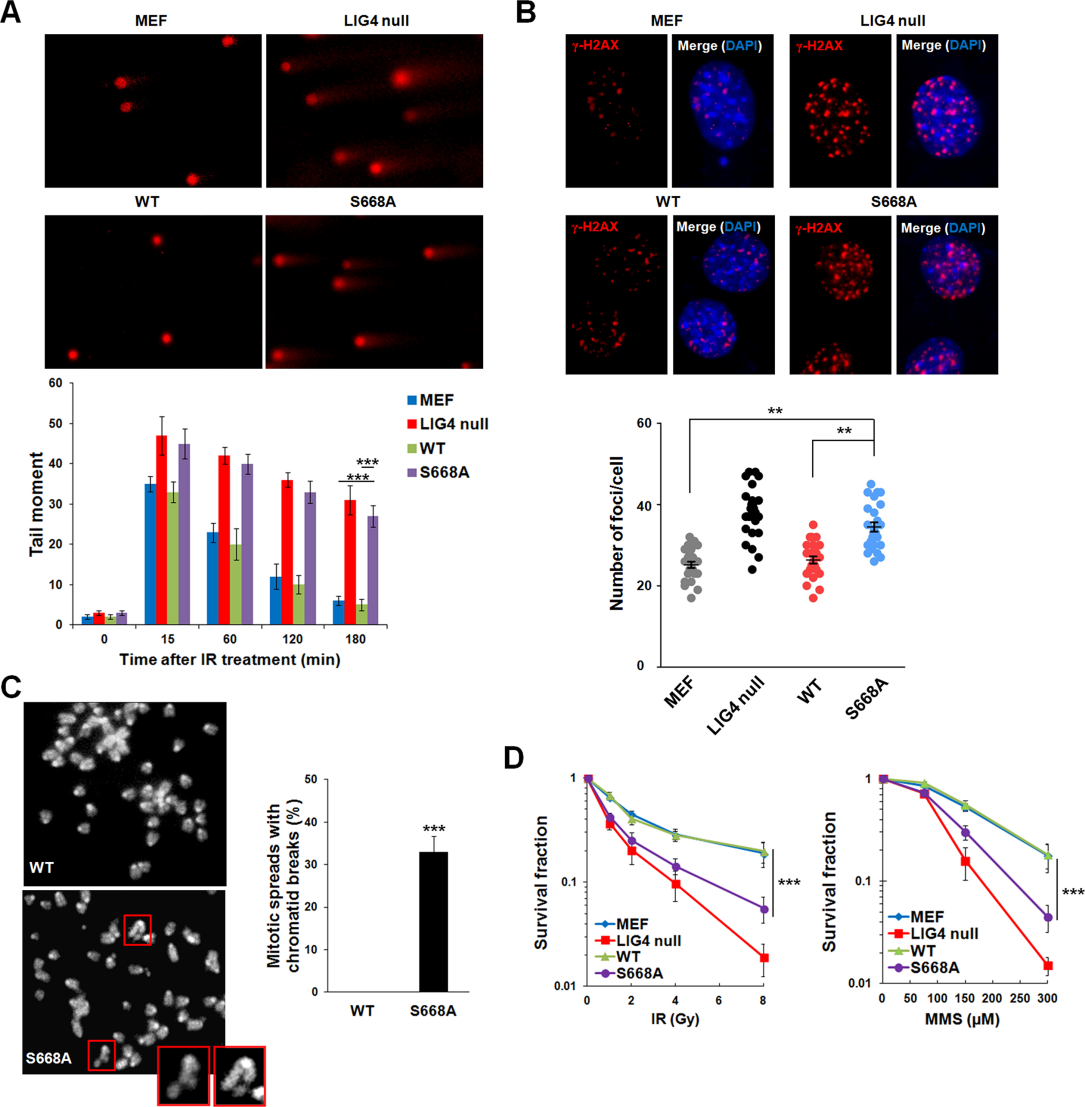
NAD^+^-mediated ligation plays an important role in DSB repair. (**A**) IR-induced DSBs is examined by neutral comet assay. Wild-type MEFs, Ligase IV^−/−^ MEFs (LIG4 null), and Ligase IV^−/−^ MEFs reconstituted with wild-type Ligase IV (WT) or the S668A mutant (S668A) were treated with IR (5 Gy) followed by 4-h recovery and neutral comet assay. Average tail moments of cells are shown in the histogram (mean ± SD, *n* = 3 independent experiments). *** denotes *P* < 0.001. (**B**) IR-induced γ-H2AX foci formation assay were analyzed by immunofluorescence method. Cells were treated with IR (2 Gy) followed by 4-h recovery and immunostained by anti-γ-H2AX antibody. Average number of foci per cell is shown in the graph. ** denotes *P* < 0.01. (**C**) Representative mitotic spreads of the WT and S668A mutant. Chromosomes were stained with DAPI. The chromatid breaks from S668A mutant are marked by red boxes. Magnified views of the breaks are displayed in boxes at the corner. Twenty mitotic spreads in each sample were examined. Percentage of chromatid breaks in each sample was summarized and presented as mean ± SD from three independent experiments. *** denotes *P* < 0.001. (**D**) Cell viability analysis was performed under different DSB agents (IR and MMS). After IR (1, 2, 4, 8 Gy) or 2-h MMS (75, 150, 300 μM) treatment, surviving cells were fixed by methanol and stained with crystal violet. Data are presented as mean ± SD (*n* = 3 independent experiments). *** denotes *P* < 0.001.

As NHEJ is one of the major DSB repair pathways, especially in G1 phase, we ask if NAD^+^-mediated DNA ligation is important for DSB repair. Ligase IV-deficient MEFs stably expressing wild-type Ligase IV or the S668A mutant were arrested in G1 phase and followed by IR treatment. With comet assays and γ-H2AX foci formation analysis, we found that DSB repair was clearly impaired in G1 cells expressing the S668A mutant (Figure 6A and B). Similar comet assay results were also observed in cells expressing K675A and N706A mutants (Supplementary Figure S10). We also treated asynchronized MEFs with IR and examined the mitotic spreads. The cells with the S668A mutant, but not the wild-type Ligase IV, harbored DSBs (Figure 6C). In addition, compared to the cells expressing wild-type Ligase IV, the cells with the S668A mutant were hypersensitive to IR or MMS retreatment (Figure 6D). Taken together, these results suggest that Ligase IV utilizes NAD^+^ for DSB repair in cells.

## DISCUSSION

In this study, we show that human DNA ligase IV is able to use NAD^+^ as an alternative co-factor for DNA ends ligation. NAD^+^ provides AMP moiety for the adenylation of Ligase IV, which triggers following steps of DNA ligation.

DNA ligase IV was first identified in HeLa cells by Lindahl laboratory. They have shown partial amino acid sequence homology with other ATP-dependent DNA ligases, including a consensus active site (54). As a result, Ligase IV was considered as an ATP-dependent DNA ligase. However, only the partial homology of the enzymatic domain may be insufficient to draw a comprehensive conclusion. Based on the known structure of Ligase IV, we analyzed the binding environment and binding modes of ATP, and docked NAD^+^ in Ligase IV catalytic cage (PDB: 3W5O (52)). The results show that Arg293 and Lys345 provide additional H-bond interactions with the NMN of NAD^+^, which enhances the affinity between NAD^+^ and the Ligase IV catalytic cage for Lys273 adenylation (Supplementary Figure S11A and B). Besides, similar to Arg293 and Lys345 of Ligase IV, two highly conserved positions of polar and positively charged amino acids are observed in the catalytic cages of other NAD^+^-dependent DNA ligases, which may serve as a molecular basis for NAD^+^-dependent adenylation (Supplementary Figure S11C). In contrast, human DNA Ligases I and III, two ATP-dependent DNA ligases, lack a significant polar residue at the similar position of Lys345 of Ligase IV (Supplementary Figure S11D). Taken together, these observations suggest that human DNA Ligase IV might be considered as an ATP/NAD^+^-mediated ligase among the human DNA ligases (Supplementary Figure S11E), and future detailed structural analyses on the catalytic domains of different ligases are needed to validate the catalytic process of the adenylation.

Ligase IV is also able to use ATP for adenylation, albeit with less efficiency compare to NAD^+^ in second round of DNA ligation. Thus, Ligase IV is very similar to the ligases found in Archaea, which use multiple sources for adenylation. We also examined if Ligase IV is able to use other nucleotide analogs including NADP, ADPr, ADP, AMP, cytidine triphosphate (CTP) and GTP. Interestingly, the ligated DNA products can be detected in the presence of ADPr, although the ligation efficiency was lower (Supplementary Figure S8). ADPr is the basic unit of poly(ADP-ribosyl)ation. Our previous study shows that the BRCT domain of Ligase IV is able to recognize ADPr, which mediates the early and fast recruitment of Ligase IV to DNA lesions. Poly(ADP-ribosyl)ation is dynamically regulated by both poly-ADP ribose polymerase (PARPs) and dePARylation enzymes such as poly-ADP ribose glycohydrolase (PARG). Massive amount of poly(ADP-ribose) is digested by PARG into ADPr within a few minutes following DNA damage at lesions. Thus, it is possible that Ligase IV is able to use ADPr to repair DNA lesions with the local high concentration of ADPr. The adenylation of Ligase IV is recycled, and Ligase IV is repeatedly used for DNA ligation. The first round of adenylation of Ligase IV is likely mediated by ATP or NAD^+^. With the higher local concentration of ADPr, it is possible that Ligase IV may use ADPr in the next round of adenylation. However, due to technical challenges, it is very difficult to examine which co-factor plays a key role in NHEJ. In particular, it is currently very difficult to distinguish NAD^+^ from ADPR in the Ligase IV-mediated repair in cells since ADPr is derived from NAD^+^-dependent PARylation. The only method to suppress the generation of ADPR but not NAD^+^ is to abolish PARG-mediated PAR degradation. However, loss of PARG will maintain the PAR chain at DNA lesions and trap DNA repair factors such as Ligase IV(28). Thus, it would be intriguing to examine the possibility of ADPr as the adenylation donor in future.

Moreover, our study suggests that the BRCT domain of Ligase IV is able to recognize NAD^+^, especially with Ser668 and Lys675 to interact with the phosphate groups in NAD^+^. The binding mode is similar to that between the phospho-peptide and the BRCT domain of BRCA1. We and others have shown that the Ser1665 and Lys1702 of the BRCA1 BRCT domain form a clamp to catch the phosphate group in a phospho-Ser motif (57–60). Here, with both Ser and Lys residues, the Ligase IV BRCT domain recognizes NAD^+^. We have also analyzed several other NAD^+^-dependent ligases and found that similar residues are conserved (Supplementary Figure S12). Thus, it is possible that other BRCT domain in the NAD^+^-dependent ligases use similar binding mode to facilitate adenylation. Of note, although Ligase III also contains a solo BRCT motif, this BRCT motif is a phospho-Ser binding module and mediates the interaction with XRCC1. Thus, Ligase III is an ATP-dependent DNA ligase.

For the DSB ligation, XRCC4, the major binding partner of Ligase IV is also required. However, the primary function of XRCC4 is to stabilize both DNA ends for facilitating the AMP moiety from 5′ end to attack 3′ OH group. XRCC4 also binds to the linker region between the tandem BRCT motifs. Although lacking XRCC4 slightly affects the adenylation efficiency, it is dispensable for the ligase-adenylate. Moreover, in the DSB ligation assay, we examined the ligation with 4 nt- or 1 nt-overhang, as ligation with overhang is more often observed during DSB repair than the blunt end ligation, and the ligation efficiency is much higher and easily measured.

Our study also shows that genetic mutation causing Ligase IV syndrome not only occurs in the enzymatic domain that impairs ligase-adenylation formation, but also is existed in the BRCT domain that abolishes the recognition of NAD^+^. Thus, our study reveals an additional pathogenic mechanism underlying the Ligase IV syndrome.

## Supplementary Material

Supplementary Data
