# Supplementary material for: Human DNA ligase IV is able to use NAD+ as an alternative adenylation donor for DNA ends ligation
Source: Nucleic Acids Res. 2018 Nov 28;47(3):1321–34. doi: 10.1093/nar/gky1202 (PMC6379666; doi:10.1093/nar/gky1202)
Supplement: Supplementary Data [file gky1202_supplemental_files.pdf]

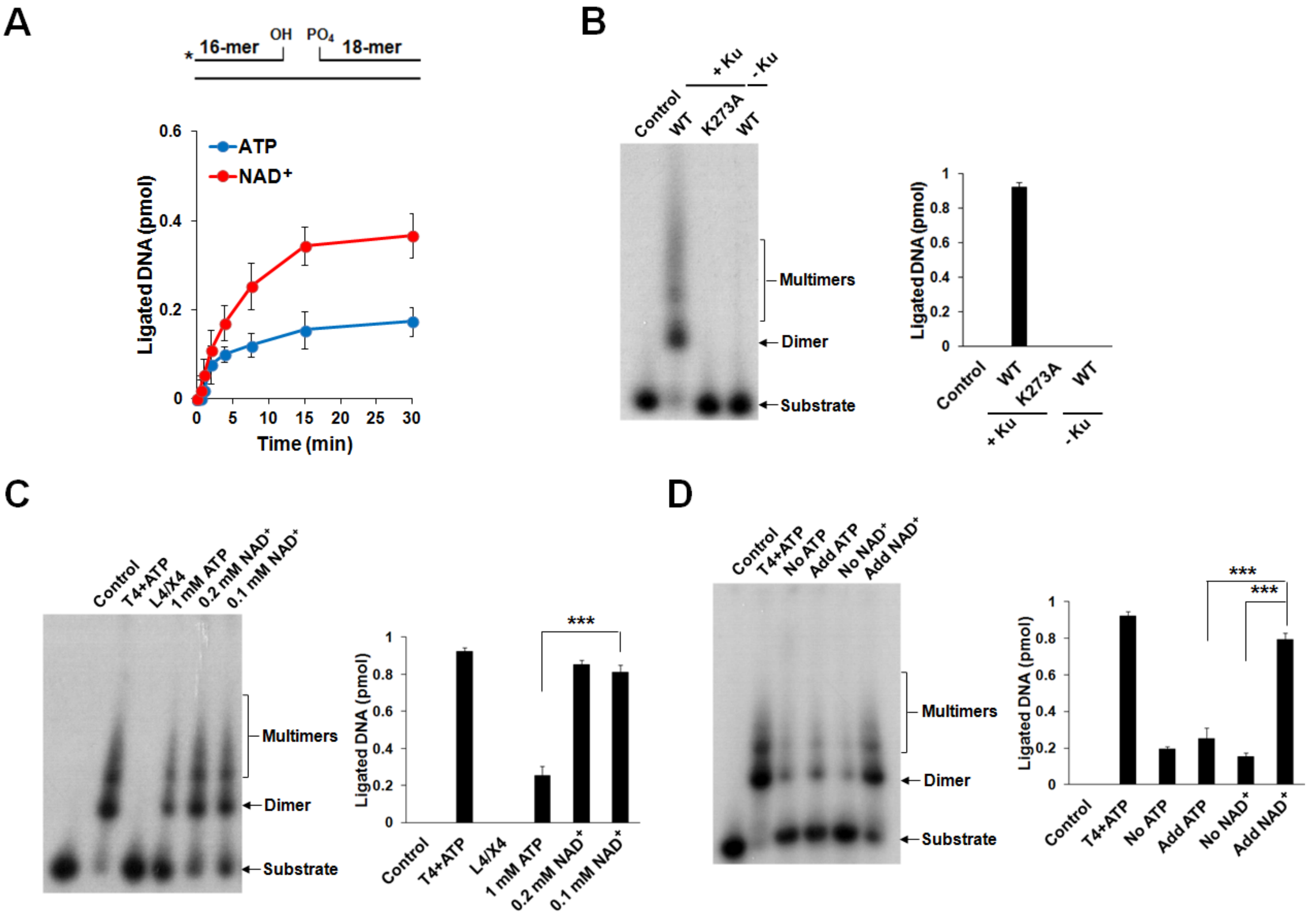

Supplementary Figure S1. DNA Ligase IV/XRCC4 uses multiple donors as co-factor for DNA ligation. (A) Time-course Nick ligation assay using 1 mM NAD^+ or ATP. (B) 4nt-overhang ligation assay using 1 mM NAD^+ with and without 25 nM Ku70/80. (C) 4nt-overhang ligation assay using 1 mM ATP and a dose course of NAD^+ as co-factor. L4/X4 means Ligase IV/XRCC4 complex without any co-factor. (D) The effect of added ATP or NAD^+ (to promote readenylation) on 4-nt overhang ligation. Reactions (20 nM Ligase IV/XRCC4 and 100 nM DNA end substrate) started with a 0.5-h incubation at 37 °C followed by addition of 1 mM ATP or 0.1 mM NAD^+, and incubation continued for 15 min at room temperature followed finally by a second incubation for 1 h. (B-D) Control means no protein and T4 DNA ligase (T4, 4000 units/mL) with ATP was used as a positive control. (A-D) Ligated products were analyzed and presented as the mean ± SD from 3 independent experiments.

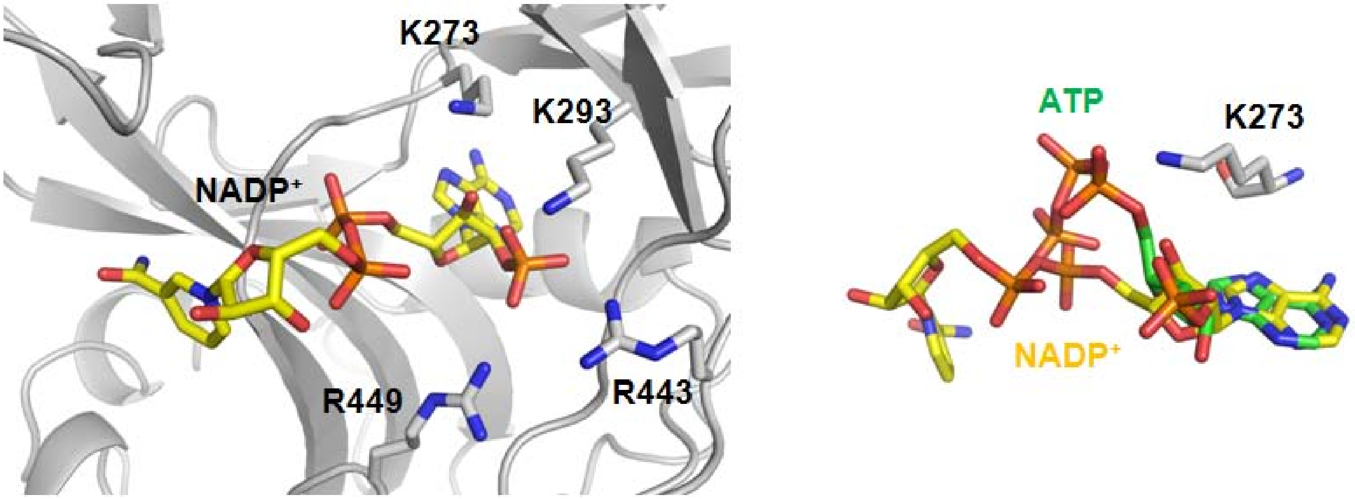

Supplementary Figure S2. $NADP^+$ is unable to be used by Ligase IV to form Ligase-adenylate. The binding mode of $NADP^+$ (yellow) in the adenylation domain of human DNA Ligase IV is different from that of ATP (green, PDB: 3W5O). AMP moieties of $NADP^+$ and ATP are not superimposed. Long distance (> 5Å) between diphosphate and Lys273 was observed, which is different from that of ATP or NAD^+ (~ 3Å from Lys273).

A

	Kinetics	
	K_m (μM)	k_{cat}/K_m ($M^{-1}S^{-1}$)
NAD^+	0.51 ± 0.12	1.7 ± 0.7×10^4

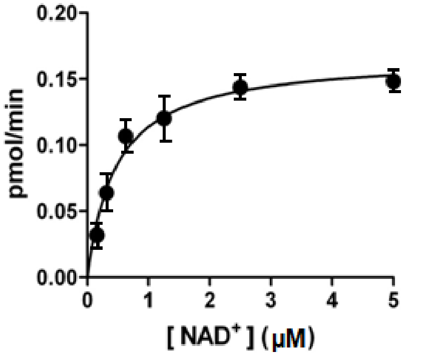

B

	Kinetics	
	$K_{d,app}$ (μM)	$k_{lig}/K_{d,app}$ ($M^{-1}S^{-1}$)
ATP	1.41 ± 0.05	9.2 ± 0.5×10^3

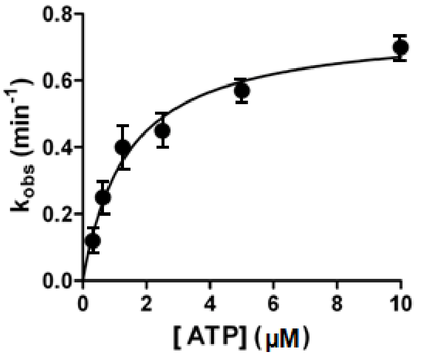

Supplementary Figure S3. Kinetic parameters of NAD^+- and ATP-mediated ligation. (A) For NAD^+-mediated ligation, 25 nM Ligase IV/XRCC4 complex was incubated with 500 nM DNA end substrates for multiple turnover analysis. The concentrations of NAD^+ were used from 0.156 μM to 5 μM. Kinetic parameters of NAD^+-mediated ligation were obtained and measured based on Michaelis-Menten equation. (B) For ATP-mediated ligation, 500 nM LigaseIV/XRCC4 complex was incubated with 100 nM DNA end substrates for single turnover analysis. The concentrations of ATP were used from 0.3125 μM to 10 μM. Saturation curve was obtained by plotting the apparent rate constant (k_{obs}) of single turnover reactions as a function of ATP concentration and fitting the data to k_{obs} = $k_{lig}[S]/(K_{d,app} + [S])$, where k_{lig} is the catalytic rate constant, and $K_{d,app}$ is the apparent equilibrium dissociation constant. (A and B) Results were analyzed from three independent experiments.

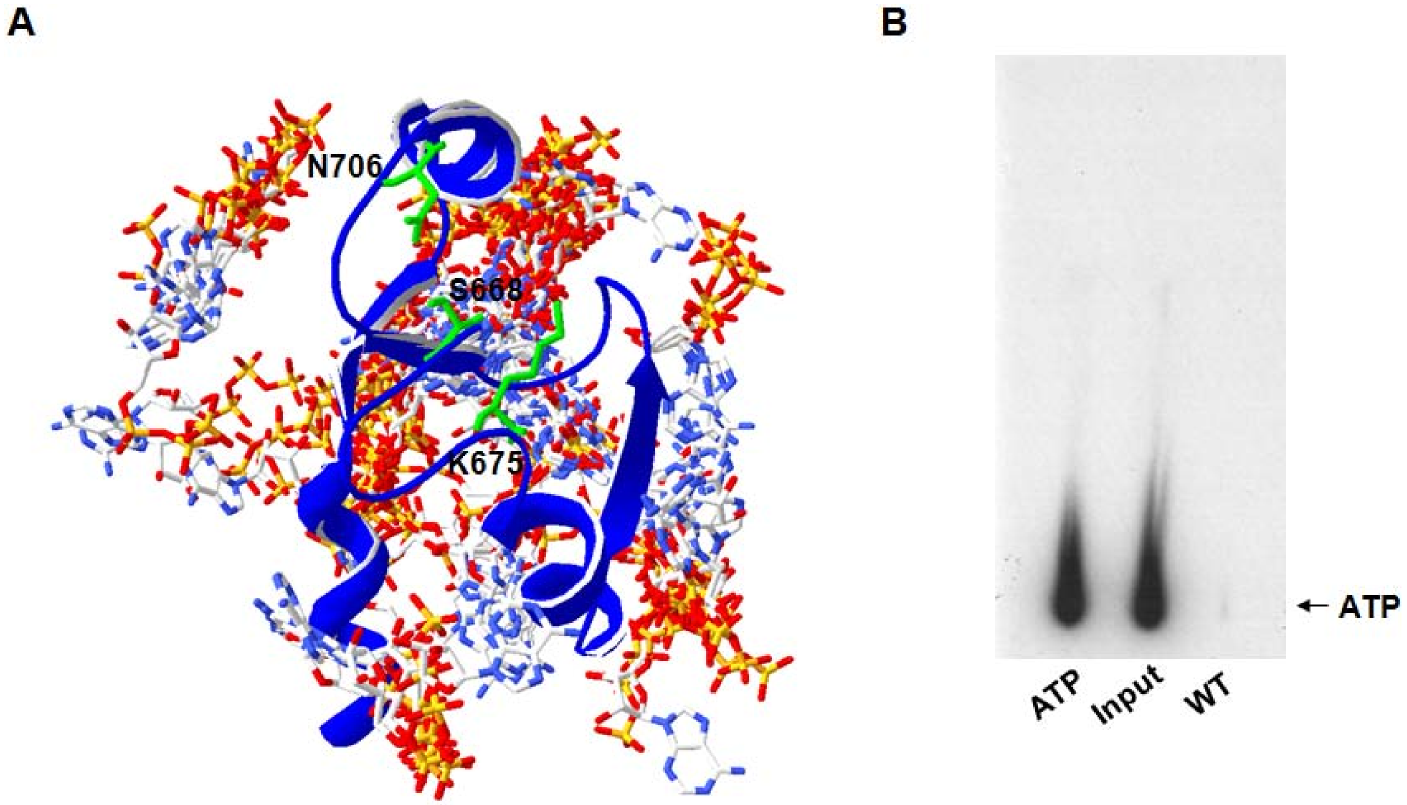

Supplementary Figure S4. The BRCT domain of Ligase IV does not bind to ATP. (A) Docking analysis using ATP and BRCT domain of Ligase IV (PDB: 2E2W). 100 binding modes of ATP were generated by GEMDOCK. Compared with NAD^+, ATP cannot fit into the pocket surrounded by S668 and K675. Thus, ATP is scattered around the BRCT domain. (B) 1 µM wild type (WT) BRCT domain were incubated with 500 nM ATP (10 nM ^{32}P-ATP and 490 nM unlabeled ATP) in the pull-down assays with 100 µl volume. The samples were analyzed by thin-layer chromatography (TLC). (n=3 independent experiments).

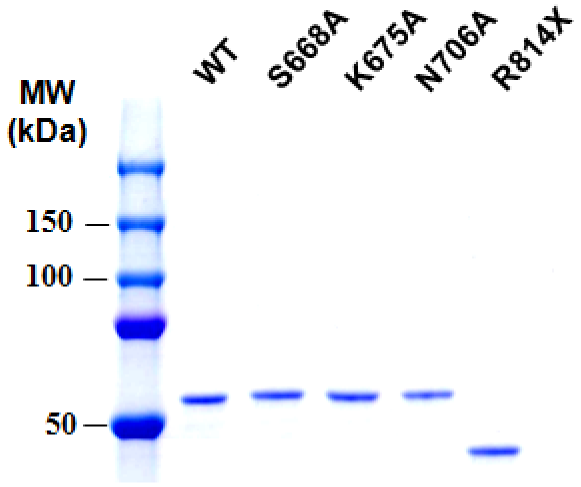

Supplementary Figure S5. Recombinant BRCT domains of DNA ligase IV. GST-wild type (WT) BRCTs and four variants (S668A, K675A, N706A, and R814X) were expressed in *E. coli*. The proteins were purified with GST beads and analyzed by 7.5 % SDS-PAGE.

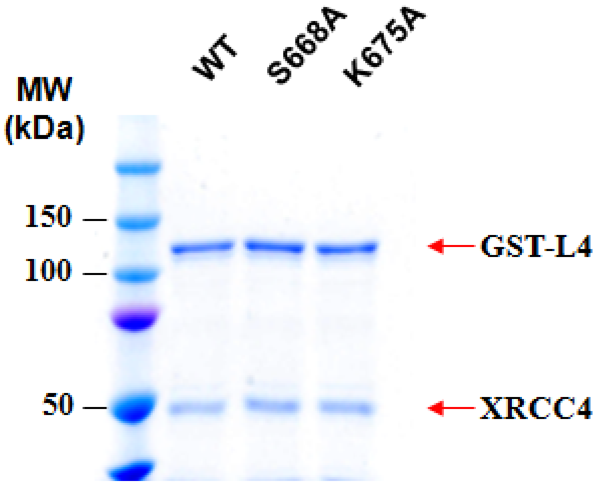

Supplementary Figure S6. Neither S668A nor K675A abolishes the interaction with XRCC4. GST- wild type (WT) Ligase IV, the S668A mutant, or the K675A mutant were co-expressed with XRCC4 (no tag) in Sf9 cells. The complex were formed and purified with GST beads. The samples were analyzed by 7.5 % SDS-PAGE.

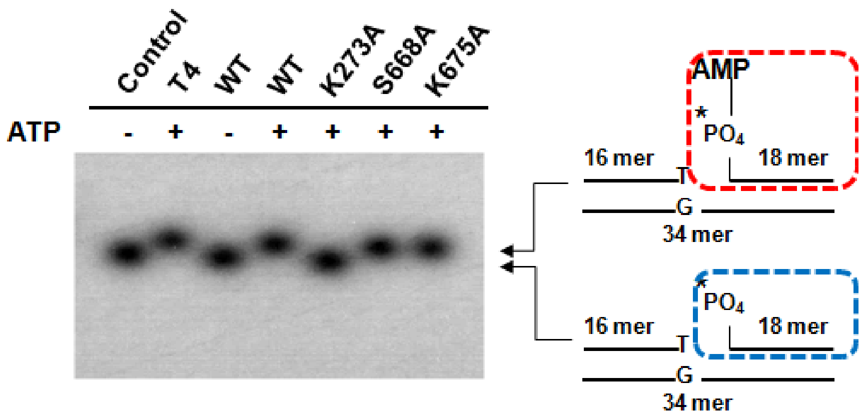

Supplementary Figure S7. Mutations of BRCT domain do not abolish formation of ATP-mediated Adenylated DNA (App-DNA). The wild type Ligase IV or its mutants (25 nM) were incubated with 1 mM ATP for adenylation, then incubated with 5 nM ^{32}P-labled 3' mismatch DNA substrates in 10 µl. A star indicates the position of the radioisotope label. App-DNA complexes were analyzed by 20 % TBE-native gel and autoradiography. Control means no protein. T4 DNA ligase (T4, 4000 units/mL) and the K273A mutant complex (K273A) were used as positive and negative control, respectively.

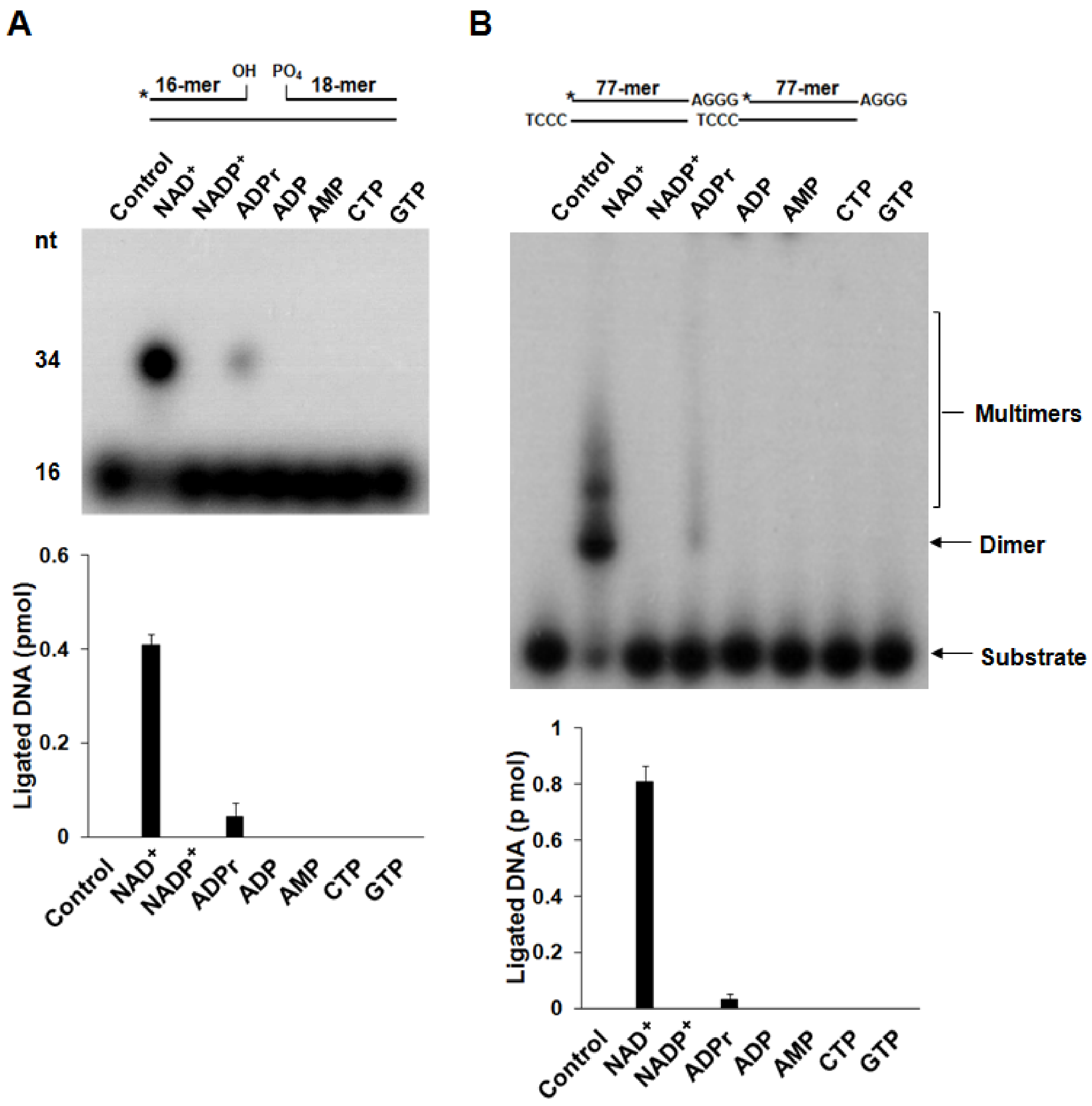

Supplementary Figure S8. Other nucleic acid-mediated ligation. 1 mM NAD^+, $NADP^+$, ADP-ribose (ADPr), ADP, AMP, CTP, and GTP were incubated with Ligase IV/XRCC4 (25 nM) for the nicked (A) or 4 nt-overhang (B) DNA ligation. The nicked DNA end substrate was 50 nM and 4 nt-overhang DNA end substrate was 100 nM. A star indicates the position of the radioisotope label. Control means no protein. NAD^+ and AMP were used as a positive control and a negative control, respectively. Ligated products were analyzed and presented as mean ± SD from 3 independent experiments.

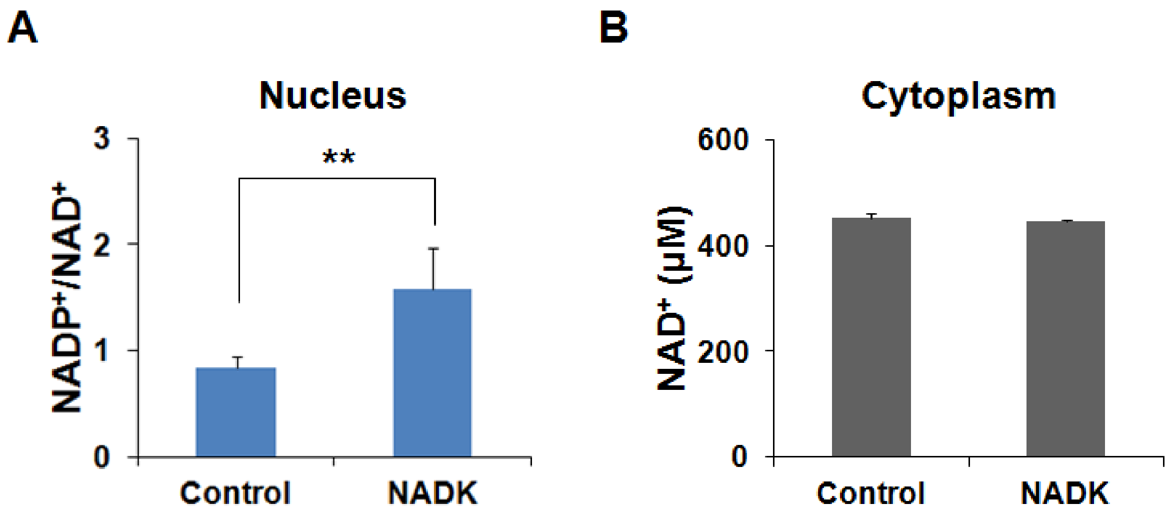

	Control	NADK
$NADP^+$ (µM)	115.1	147.3
NAD^+ (µM)	138.3	93.5

Supplementary Figure S9. NADK changes the ratio of nuclear $NADP^+/NAD^+$, but the cytoplasmic NAD^+ level is unaffected. (A) The $NADP^+/NAD^+$ ratio were measured by EnzyChrom assay kits with nucleus extracts from U2OS (control) and U2OS-NADK cells. Bar graph shows the mean $NADP^+/NAD^+$ ratio (n=3 independent experiments, ** denotes $p < 0.01$). (B) The concentrations of NAD^+ in cytoplasm were examined by EnzyChrom NAD^+/NADH assay kits. Bar graph shows the mean concentration of NAD^+ (n=3 independent experiments).

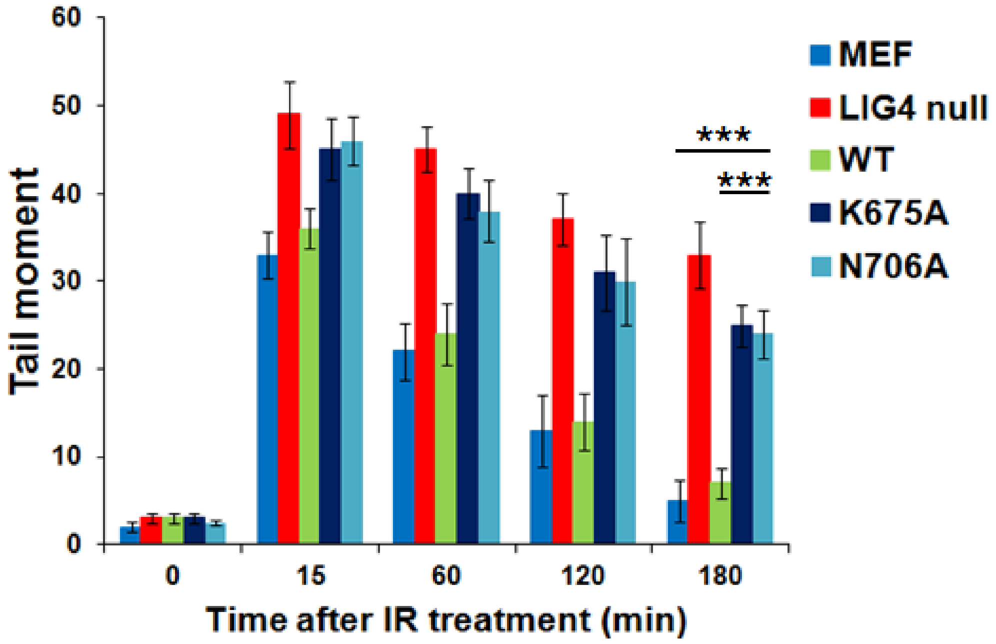

Supplementary Figure S10. Mutations of BRCT domain impair NAD^+-mediated DSB repair in cells. IR-induced DSBs is examined by neutral comet assay. Wild type MEFs, Ligase $IV^{-/-}$ MEFs (LIG4 null), and Ligase $IV^{-/-}$ MEFs reconstituted with wild type Ligase IV (WT) or two mutants (K675A and N706A) were treated with IR (5 Gy) followed by 4-hour recovery and neutral comet assay. Average tail moments of cells (mean ± SD) were shown in the histogram from 3 independent experiments. *** denotes $p < 0.001$.

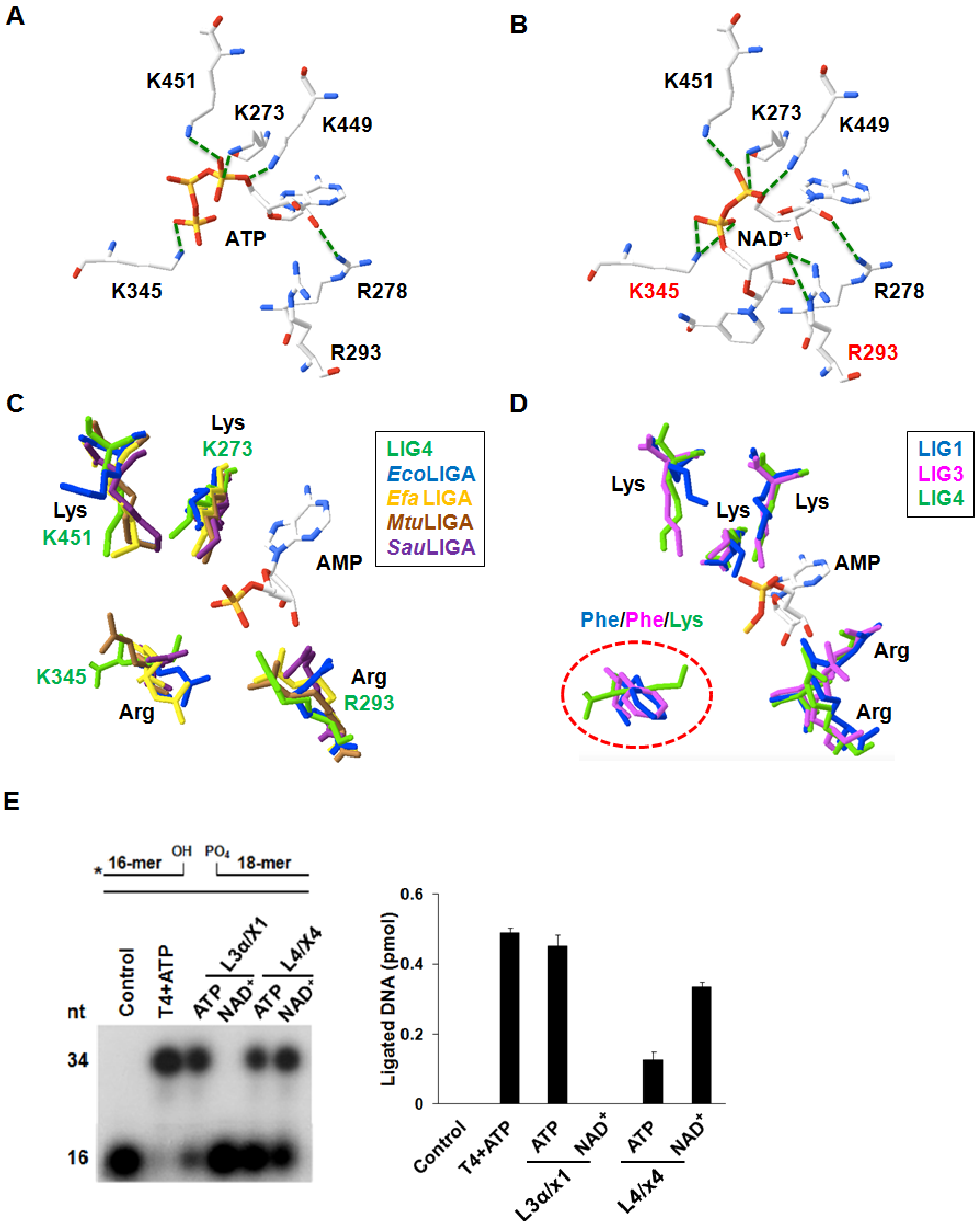
A
K451
K273
K449
ATP
K345
R278
R293
B
K451
K273
K449
NAD+
K345
R278
R293
C
Lys
K273
LIG4
EcoLIGA
Efa LIGA
MtuLIGA
SauLIGA
Lys
K451
AMP
K345
Arg
Arg
R293
D
LIG1
LIG3
LIG4
Lys
Lys
Lys
AMP
Phe/Phe/Lys
Arg
Arg
E
* 16-mer OH PO4 18-mer
nt
Control
T4+ATP
L3α/X1
ATP
NAD+
L4/X4
ATP
NAD+
34
16
Ligated DNA (pmol)
0.6
0.4
0.2
0
Control
T4+ATP
ATP
NAD+
ATP
NAD+
L3α/x1
L4/x4

Supplementary Figure S11. Comparisons of active sites of Ligase IV and other NAD^+-mediated DNA ligases. The binding modes of ATP (A) and docked NAD^+ (B) in the Ligase IV active site (PDB: 3W5O). Hydrogen bounds are shown in green dotted lines. The key residues of Ligase IV for NAD^+-mediated adenylation are highlighted in red. (C) Superimposition of Ligase IV (PDB: 3W5O) and four NAD^+-dependent ligases (PDBs: 2OWO, 1TAE, 1ZAU, and 3JSN) from *Escherichia coli* (*Eco*), *Enterococcus faecalis* (*Efa*), *Mycobacterium tuberculosis* (*Mtu*), and *Staphylococcus aureus* (*Sau*). (D) Overlap of three human DNA ligases (LIG1: 1X9N; LIG3: 3L2P; LIG4: 3W5O) for active sites analysis. The key different residues are marked by red dotted circle. (E) Nicked ligation assays were carried out with 25 nM Ligase III-α/XRCC1 (L3α/X1) complex and Ligase IV/XRCC4 (L4/X4) complex together with 1 mM ATP or NAD^+. Control means no protein and T4 DNA ligase (T4, 4000 units/mL) with ATP was used as a positive control. Ligated products were analyzed and presented as mean ± SD from 3 independent experiments.

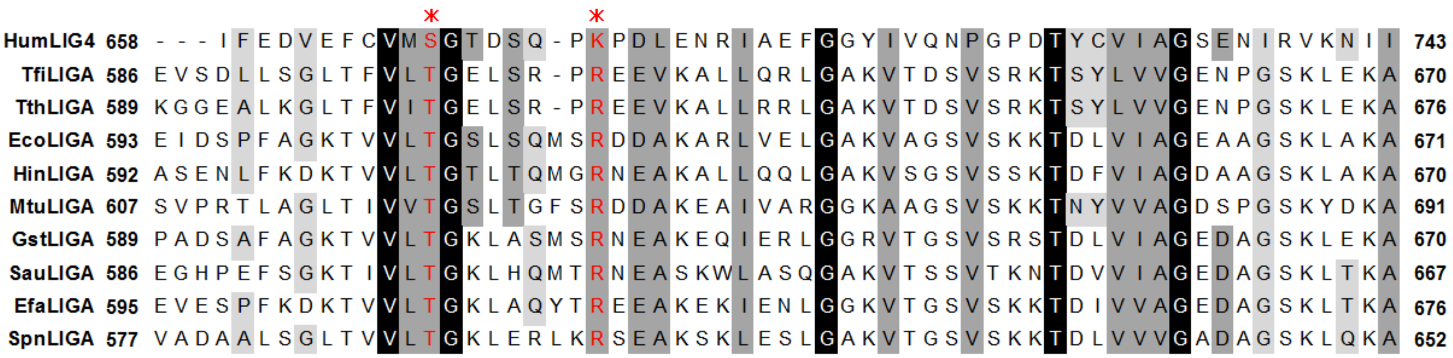

Supplementary Figure S12. Alignment of amino acid sequence similarity of human DNA ligase IV BRCT1 and other NAD$^+$-dependent DNA ligase BRCT domains. Similar residues (marked in red) that form the potential phosphate group binding pocket are observed in the BRCT domain of human DNA ligase IV and nine other NAD$^+$-dependent DNA ligases including *Thermus filiformis* (*Tfi*), *Thermus thermophilus* (*Tth*), *Escherichia coli* (*Eco*), *Haemophilus influenza* (*Hin*), *Mycobacterium tuberculosis* (*Mtu*), *Geobacillus stearothermophilus* (*Gst*), *Staphylococcus aureus* (*Sau*), *Enterococcus faecalis* (*Efa*), and *Streptococcus pneumoniae* (*Spn*). Identical amino acids and similar residues are highlighted on a shaded background. Dashes indicate gaps in the alignments. Stars denote the amino acid residues mutated for DNA ligase IV in this study.
